# Electrode-Free ECG Monitoring with Multimodal Wireless Mechano-Acoustic Sensors

**DOI:** 10.3390/bios15080550

**Published:** 2025-08-20

**Authors:** Zhi Li, Fei Fei, Guanglie Zhang

**Affiliations:** 1 College of Computer Science and Software Engineering, Shenzhen University, Shenzhen 518060, China; 2 College of Automation Engineering, Nanjing University of Aeronautics and Astronautics, Nanjing 211100, China; 3 Department of Mechanical Engineering, City University of Hong Kong, Hong Kong, China; 4 City University of Hong Kong Shenzhen Research Institute, Shenzhen 518057, China

**Keywords:** electrocardiogram (ECG), seismocardiography (SCG), phonocardiogram (PCG), mechano-acoustic sensors, electrode-free ECG monitoring, Internet-of-Medical-Things (IoMT)

## Abstract

Continuous cardiovascular monitoring is essential for the early detection of cardiac events, but conventional electrode-based ECG systems cause skin irritation and are unsuitable for long-term wear. We propose an electrode-free ECG monitoring approach that leverages synchronized phonocardiogram (PCG) and seismocardiogram (SCG) signals captured by wireless mechano-acoustic sensors. PCG provides precise valvular event timings, while SCG provides mechanical context, enabling the robust identification of systolic/diastolic intervals and pathological patterns. A deep learning model reconstructs ECG waveforms by intelligently combining mechano-acoustic sensor data. Its architecture leverages specialized neural network components to identify and correlate key cardiac signatures from multimodal inputs. Experimental validation on an IoT sensor dataset yields a mean Pearson correlation of 0.96 and an RMSE of 0.49 mV compared to clinical ECGs. By eliminating skin-contact electrodes through PCG–SCG fusion, this system enables robust IoT-compatible daily-life cardiac monitoring.

## 1. Introduction

Cardiovascular diseases account for approximately 32% of global mortality and require continuous monitoring with high diagnostic accuracy [1,2]. Detecting critical events such as arrhythmias, ischemic changes, and HRV anomalies requires accurate, continuous monitoring. Traditional methods such as periodic clinical visits and Holter monitors offer limited snapshots of cardiac health, often missing critical transient events such as arrhythmias or ischemic episodes. The Internet-of-Things (IoT) paradigm presents a transformative solution, enabling pervasive, real-time, and longitudinal monitoring of vital signs outside clinical settings. Wireless sensors can continuously capture electrocardiogram (ECG), photoplethysmogram (PPG), blood pressure, and activity data [3]. Nevertheless, achieving the diagnostic accuracy necessary for clinical decision-making in ambulatory environments remains a significant challenge. While an ECG remains the gold standard for cardiac assessment, leveraging IoT technologies and advanced algorithms shows great promise. These innovations can improve disease diagnosis based on biomedical signals. For the ECG measurement method, traditional adhesive electrodes face critical limitations in IoT-enabled healthcare. Prolonged skin–electrode contact disturbs the electrochemical equilibrium at the interface, triggering redox reactions that destabilize impedance. Concurrently, sweat-induced capacitance variations reduce the signal-to-noise ratio (SNR), compromising signal fidelity [4,5]. These issues are further exacerbated by the wearability challenges of IoT-enabled healthcare, including skin irritation and progressive performance deterioration, which limit the long-term practicality of ECG electrodes [6,7]. This motivates research into electrode-free sensing for monitoring cardiac activities.

To address these challenges, researchers have pursued alternative sensing modalities that circumvent direct skin contact while preserving diagnostic fidelity. Capacitively coupled electrodes emerged as an early solution, leveraging ultra-high impedance circuits to detect cardiac electrical activity through insulating materials such as clothing [8]. However, their performance degrades markedly during physical activity due to unstable electrode–skin coupling distances and susceptibility to electromagnetic interference. Flexible epidermal electronics represent another paradigm, employing ultrathin, stretchable sensors to conform seamlessly to skin morphology. Recent innovations, such as the motion-unrestricted dynamic electrocardiogram (MU-DCG) system [9], minimized discomfort compared to Holter monitors with a pressure-activated flexible skin socket. Optical sensing via photoplethysmography is a low-cost alternative commonly used in wearable devices for heart rate monitoring, but it primarily measures blood volume changes rather than the full PQRST waveforms seen in an ECG [10].

The cardiac excitation–contraction coupling (ECC) mechanism offers a promising pathway to bridge cardiac electrical activity and mechanical motion [11,12]. During each heartbeat, electrical impulses trigger synchronized myocardial contractions, generating subtle mechanical vibrations on the chest wall. Recent advances in sensor technology have enabled indirect ECG estimation by capturing these vibrations via modalities such as seismocardiography (SCG) [13] and millimeter-wave radar [14]. However, existing approaches face critical barriers to widespread adoption: radar-based systems require bulky hardware and are sensitive to environmental interference, while SCG often struggles with motion artifacts and inter-subject variability. Therefore, overcoming these limitations in hardware robustness, noise interference, and signal reliability is crucial for the practical clinical application of vibration-based ECG monitoring.

In this work, we propose a wireless, electrode-free ECG estimation system that integrates synchronized SCG and PCG signals to reconstruct ECG waveforms without direct skin contact electrodes. By leveraging the physiological coupling between cardiac electrical and mechanical events, we design a multimodal deep learning framework combining long short-term memory (LSTM) networks with a transformer architecture. The model learns to correlate mechanical and acoustic cardiac signatures—capturing both myocardial dynamics and valvular timing—to produce accurate ECG waveform estimations. This approach offers a wearable, noninvasive, and scalable solution for continuous cardiac monitoring in IoT healthcare systems.

The remainder of this paper is organized as follows: Section 2 reviews related work in ECG monitoring and deep learning algorithms. Section 3 details the system design and methodology. Section 4 presents experimental results and Section 5 comprises discussions, while Section 6 concludes this paper and outlines future directions.

## 2. Related Work

ECG monitoring has evolved significantly, driven by advancements in sensor technology and computational methods. Traditional electrode-based systems remain the clinical gold standard due to their high diagnostic accuracy [15]. However, their reliance on adhesive electrodes introduces challenges such as skin irritation, motion artifacts, and limited long-term usability, particularly for ambulatory monitoring. To address these issues, wearable ECG devices such as chest straps and smartwatches have emerged, offering continuous monitoring with improved user comfort.

### 2.1. Cardiac Excitation–Contraction Coupling (ECC)

Cardiac excitation–contraction coupling (ECC) describes the physiological mechanism through which electrical impulses in the heart initiate mechanical responses that drive blood circulation. This complex process begins with the generation and propagation of electrical signals across the atria and ventricles. These signals appear as the P wave, QRS complex, and T wave on the electrocardiogram (ECG), reflecting atrial depolarization, ventricular depolarization, and repolarization, respectively. The electrical activity stimulates calcium ion release within cardiac myocytes, triggering the contraction and mechanical movement of the heart walls and valves. These mechanical events are captured using noninvasive sensing modalities. Phonocardiography (PCG) records heart sounds, particularly the S1 and S2 sounds corresponding to mitral and aortic valve closure.

These sounds are generally aligned with the QRS complex and the end of the T wave. SCG detects micro-vibrations on the chest surface, providing markers such as aortic valve opening (AO) and closure (AC), which offer precise mechanical timing cues. Together, PCG and SCG offer complementary insights into cardiac dynamics, capturing valve activity and myocardial motion. This mechano-acoustic information can be used to estimate underlying electrical signals, enabling ECG reconstruction in scenarios where direct electrode contact is undesirable or impractical [16,17].

### 2.2. Deep Learning for ECG Synthesis and Generation

Deep learning has emerged as a powerful tool to address data scarcity and domain shifts in healthcare applications. Generative deep learning has become a standard approach for synthesizing ECG signals. Recurrent or convolutional generative adversarial networks (GANs) are used to model ECG morphology. Zhu et al. [18] proposed a BiLSTM–CNN GAN that generates synthetic ECG beats with high morphological fidelity. Beyond GAN models, hybrid CNN–transformer models capture long-range dependencies across entire cardiac cycles. Xia et al. [19] introduced a transformer-enhanced GAN (TCGAN) that improved classification accuracy on imbalanced datasets. More recent GAN variants, such as CycleGAN, conditional GANs, and auxiliary conditional GANs, have been applied for ECG augmentation. Chen et al. [20] translated PCG signals to ECG waveforms using a CycleGAN framework. These studies demonstrate the promise of learning cross-modal mappings, but they either focus on single modalities or emphasize data augmentation rather than direct ECG reconstruction [21,22,23].

Recent efforts have focused on electrode-free methods to monitor cardiac activity by leveraging indirect physiological signals. Radar systems have been explored to capture chest micro-motions noninvasively [14], although they require bulky hardware and are vulnerable to environmental noise. SCG and gyrocardiography (GCG) measure chest wall vibrations caused by cardiac mechanical activity [24]. Parlato et al. achieved high-accuracy heartbeat detection from SCG signals under controlled conditions [25]. Tapotee et al. introduced M2ECG, a deep learning model that translates SCG and GCG signals into ECG waveforms [26]. However, their approach does not incorporate valve timing cues available from phonocardiography. Our method addresses this limitation by combining SCG and PCG in a hybrid LSTM–transformer architecture. This multimodal fusion captures complementary mechanical and acoustic information, resulting in more accurate and robust ECG reconstruction.

## 3. Methodology

**Figure 1:**
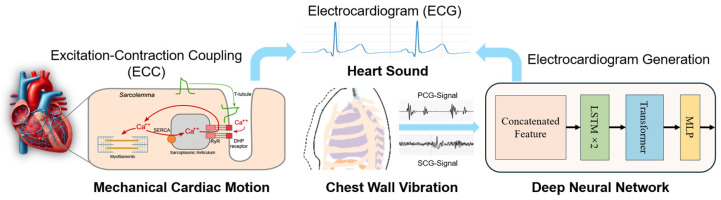
Electrode-free ECG monitoring with the ECC mechanism.

The coupling of cardiac excitation and contraction is a dynamic interaction between electrical signals and mechanical responses. PCG provides noninvasive auditory tracking of valve closure events (S1/S2), while SCG captures kinetic vibrations reflecting myocardial wall motion (AO/AC). This synergy enables ECG reconstruction by mapping mechano-acoustic signatures to underlying electrical activity through excitation–contraction coupling principles. MEMS inertial and microphone sensors provide unique and powerful means to capture the mechanical consequences of this coupling, allowing for the noninvasive, real-time monitoring of the mechanical components of the cardiac cycle. Based on the strong correlation between cardiac mechanical and electrical signals, we will realize the conversion of cardiac mechanical signals into cardiac electrical signals through deep networks. This conversion from mechanical sensing to an electrical presenting capability not only enhances our understanding of normal cardiac function but also holds great promise for the detection and management of cardiac pathologies. The proposed electrode-free ECG monitoring system integrates wireless mechano-acoustic sensors, a deep learning framework grounded in cardiac biophysics, and IoT-enabled real-time analysis, as described in Figure 1. Moreover, the sensitivity of MEMS sensors to the minute movements of the chest wall enables the detection of subtle changes in mechanical activity that might not be visible through conventional methods such as ECG alone. This makes MEMS inertial sensors especially useful in monitoring conditions where the heart’s electrical and mechanical activities become uncoupled, such as in heart failure or ischemic heart disease. By assessing both electrical signals and mechanical responses, MEMS sensors offer a more holistic view of cardiac health, paving the way for more sophisticated, wearable diagnostic tools.

This section elaborates on the system architecture, training a hybrid LSTM–transformer model on synchronized ECG and MEMS accelerometer sensor recordings, and the implementation details with the multi-scale wavelet-optimized loss function. The integration of the MEMS accelerometers into the monitoring of cardiac function offers significant advantages, particularly in noninvasive continuous monitoring. The data provide real-time feedback on the mechanical responses of the heart without the need for direct intervention, and when used in combination with an IoT connection, it can provide a more comprehensive understanding of both electrical and mechanical heart functions. In clinical settings, this dual-sensing approach holds promise for the early detection of cardiac abnormalities, offering more accurate diagnostics and potentially improving patient outcomes by enabling timely interventions.

### 3.1. System Design and Hardware of the Measurement Device

#### 3.1.1. Internet of Medical Things (IoMT) Architecture

**Figure 2:**
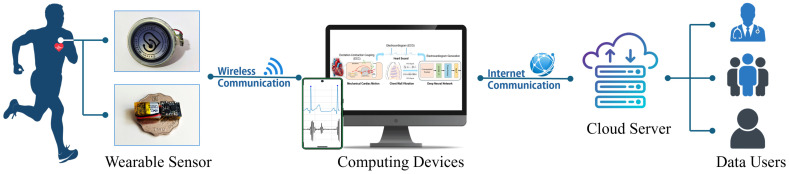
IoMT-based electrode-free ECG monitoring system.

The Internet of Medical Things (IoMT) constitutes a specialized healthcare subset of the broader IoT ecosystem, while IoTs are general-purpose networks of interconnected physical devices and systems. The IoMT refers to a network of connected medical devices, software applications, and healthcare systems that enable the real-time collection, transmission, and analysis of patient data. Figure 2 illustrates the proposed IoMT-based ECG monitoring framework. A lightweight sensor node hardware integrates a MEMS microphone and accelerometer to acquire synchronized PCG and SCG signals. These recordings are wirelessly transmitted via Bluetooth Low Energy or Wi-Fi to a proximal edge device, such as a smartphone or dedicated gateway.

At the edge, incoming data undergo initial preprocessing, including bandpass filtering to suppress noise, amplitude normalization, and segmentation into fixed-length windows. This local processing reduces the data volume and ensures that only the most relevant information is forwarded. The edge device may also perform simple feature extraction or heartbeat detection to support immediate, low-latency feedback. The processed data are then transmitted to the cloud server, which serves as the central computing hub. In the cloud, deep learning models such as the hybrid LSTM–transformer network reconstruct the ECG waveform from the synchronized PCG and SCG inputs. The cloud infrastructure also supports long-term data storage, advanced analytics, and clinician-facing dashboards. Historical data can be reviewed for diagnostic insights, and model updates can be deployed seamlessly.

By distributing tasks between the sensor node, edge computing device, and cloud server, the system strikes a balance between responsiveness and scalability. This architecture eliminates the need for intermediate fog nodes, simplifying deployment while maintaining robust performance for continuous, real-time cardiac monitoring applications.

#### 3.1.2. Hardware Design of Mechano-Acoustic Sensors

The design of the hardware of the mechano-acoustic sensors of the wearable device is meticulously crafted to ensure optimal performance, energy efficiency, and the seamless integration of various components. At the heart of the system lies the ESP32 Wi-Fi and Bluetooth SoC (system-on-chip) [27], which serves as the central processing unit responsible for sensor data collection, wireless communication, and peripheral interactions. This dual-core processor is designed to balance high performance with low power consumption, making it ideal for wearable applications where energy efficiency is critical.

**Figure 3:**
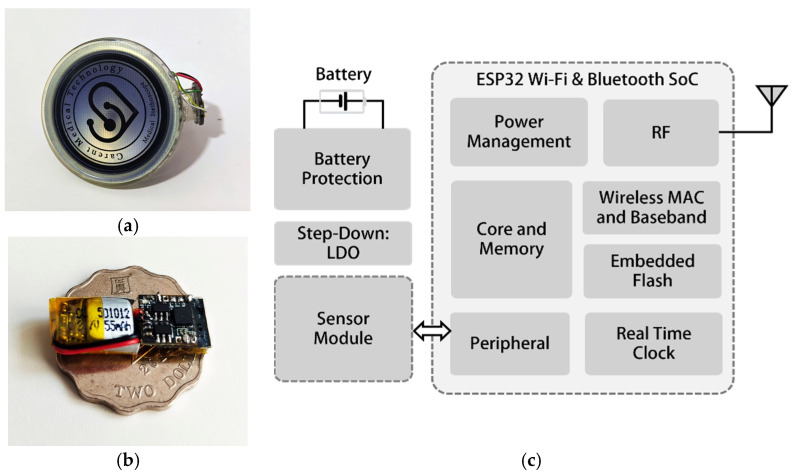
Hardware design of mechano-acoustic sensor nodes. (**a**) Digital stethoscope with MEMS microphone sensor; (**b**) vibration device with MEMS accelerometer; (**c**) block diagram of wireless sensor nodes.

As illustrated in Figure 3a,b, the mechano-acoustic sensors consist of a MEMS accelerometer and MEMS microphone mounted on a lightweight chest patch. Figure 3c presents the block diagram of the wireless sensor node, highlighting the ESP32 SoC, power management, real-time clock, and memory subsystems. The patch integrates a 3-axis accelerometer and microphone for mechano-acoustic sensing. At its core sits an ESP32 SoC with dual-core Wi-Fi/Bluetooth for on-board data acquisition and wireless transmission. Power management relies on low-dropout regulators and battery protection circuitry to maintain stable voltages, prevent overcharge/discharge, and extend battery life. To address critical power constraints in wearable IoMT applications, the wireless sensor node implements three optimized operational modes, including a 30 mA data acquisition mode during mechano-acoustic sensing, a 100 mA burst transmission mode for compressed wireless data transfer, and a 1 mA sleep mode with peripheral shutdown. Through adaptive power gating and duty cycling, the system supports hourly 1 min monitoring sessions over 24 h of continuous operation using a 55 mAh lithium-ion battery. This power-optimized architecture focuses exclusively on reliable physiological data capture and transmission, while computationally intensive tasks, including signal processing and algorithm inference, are handled by dedicated external computing devices. A real-time clock ensures precise timestamping for synchronized PCG, SCG, and ECG data logging. An embedded flash memory stores firmware and temporary data. This modular design balances low power consumption, high performance, and user comfort for long-term cardiac monitoring applications.

### 3.2. Preprocessing for Chest Wall Vibration and Acoustic Signals

The raw SCG signal has large gravity and motion components, since cardiac vibrations measurable at the chest wall exhibit extremely low amplitudes of 0.2–0.5 mm and occupy a high-frequency band from roughly 5 to 100 Hz [28]. In contrast, the static gravitational acceleration (g) of 9.81 m/s^2^ and low-frequency artifacts induced by respiration and body motion are orders of magnitude larger in amplitude. Consequently, these motion artifacts typically dominate the raw accelerometer signal, obscuring the underlying cardiac components. To recover the waveform of cardiac vibrations, the preprocessing pipeline first applies geometrically consistent gravity compensation to remove constant biases and the gravitational component. Subsequently, high-pass filtering is employed to eliminate slow drift while preserving the high-frequency cardiac signal content.

Initially, the tri-axial accelerometer raw data are calibrated by removing fixed sensor offsets. After offset correction, the accelerometer output under static conditions equals the true gravity vector projected onto each sensor axis. Using this calibrated data, we compute the instantaneous roll (φ) and pitch (θ) angles from the gravity vector alone. The gravity projections satisfy the following:

(1) $$a_{x}=g\sin\;\theta ,\;a_{y}=-g\sin\;\phi \cos\theta ,\;a_{z}=-g\cos\;\phi \cos\theta$$

Therefore, the estimation of orientation with tilt angles based on the projection of gravity onto the axes can be derived from the Euler rotation convention as follows:

(2) $$\theta =asin\;\left(\frac{a_{x}}{g}\right),\;\phi =atan2\;\left(a_{y},\;a_{z}\right)\;$$

where $a_{x},\;{\;a}_{y},\;\;a_{z}$ are the calibrated tri-axial accelerometer output data.

To smooth the tilt estimates over time, a Kalman filter is applied independently to each angle. We use a one-dimensional state for ϕ and θ with the prediction model. The measurements of angles at each sample are the instantaneously computed angles from the accelerometer.

Using the filtered roll and pitch, we form a rotation matrix that maps sensor-frame accelerations into a world frame in which the *Z*-axis aligns with gravity. Denote $R_{x}\left(\phi \right)$ and $R_{y}\left(\theta \right)$ as the rotation matrices about the sensor’s X and Y axes (with Z fixed).

(3) Rxϕ=1000cos ϕsin ϕ0−sin ϕcos ϕ, Ryθ=cos θ0−sin θ010sin θ0cos θ

Once the orientation is estimated, we form the rotation matrix to project the gravity vector into the sensor frame. We then compute the rotation matrix $R=R_{y}\left(\theta \right)\;R_{x}\left(\phi \right)$ to align the sensor frame with gravity, which transforms the vector $\vec{a_{s}}={\left[a_{x},a_{y},a_{z}\right]}^{T}$ from sensor coordinates into the earth-fixed world frame: $\vec{a_{w}}=R\;\vec{a_{s}}$. In this world frame, we therefore subtract ${\left[0,0,g\right]}^{T}$ to remove the gravitational component:

(4) $${\vec{a}}_{w}^{*}=R{\left[a_{x},a_{y},a_{z}\right]}^{T}-{\left[0,0,g\right]}^{T}$$

The gravity-compensated vector’s representation in the sensor frame is ${\vec{a}}_{s}^{*}=R^{-1}{\vec{a}}_{w}^{*}$. This geometric gravity compensation ensures that only true dynamic accelerations from respiration, motion, and cardiac beating remain, without residual tilt-induced cross-terms. Because it uses the full 3D orientation, gravity is removed accurately even if the chest tilts, preserving the orientation of the cardiac signal components.

The raw PCG audio was captured at 48 kHz in WAV format and first passed through a bandpass filter between 20 and 200 Hz to isolate heart sound frequencies and suppress low-frequency respiratory noise and high-frequency artifacts. After filtering, the waveform was downsampled to 250 Hz to match the SCG sampling rate, ensuring synchronous multimodal inputs. We then applied a moving-average envelope detector to the downsampled PCG to accentuate S1 and S2 valve closures, followed by segment-wise standardization to normalize amplitude variations across subjects and recording sessions. Finally, each PCG segment was visually inspected and aligned with its corresponding SCG and ECG windows via shared timestamps. This preprocessing yields orientation-invariant, noise-reduced PCG inputs that complement the gravity-compensated, bandpass filtered SCG signals in the deep learning model, enabling robust fusion of acoustic and vibrational cardiac features.

### 3.3. Deep Network with Hybrid LSTM–Transformer Architecture

In this work, we propose a unified deep learning model designed to translate synchronized phonocardiogram and seismocardiogram inputs into reconstructed electrocardiogram waveforms. Our architecture combines the strengths of long short-term memory (LSTM) networks for capturing local temporal dependencies with the global contextual modeling capabilities of transformer-based self-attention mechanisms. This hybrid LSTM–transformer framework enables robust, end-to-end mapping from mechano-acoustic signals to the target ECG signal.

#### 3.3.1. LSTM Encoder and Transformer-Based Contextual Refinement

The model processes two parallel input streams with a time series of acoustic recordings and a time series of vibrational signals, both preprocessed and sampled at 250 Hz. These streams are concatenated at the feature dimension. This early fusion strategy ensures that the network learns cross-modal correlations from the initial encoding stage.

The fused input is first passed through a shared LSTM encoder comprising two stacked bidirectional LSTM layers, each with 128 hidden units per directional flow. Bidirectionality allows the network to capture dependencies both before and after each time step, which is critical for aligning PCG acoustic events (e.g., S1/S2 sounds) with corresponding ECG features (e.g., QRS onset and T-wave peaks). A dropout rate of 0.2 is applied after each LSTM layer to mitigate overfitting and improve generalization across subjects.

Following the LSTM encoder, the output sequence undergoes a dimensionality projection into a higher-dimensional embedding space via a linear layer. Positional encodings—sinusoidal functions of the time index—are added to these embeddings to retain temporal order information, which is not inherently present in the transformer’s self-attention mechanism. The enhanced embeddings then enter a transformer encoder module composed of four identical layers, each containing:

- Multi-head self-attention block: Parallel attention heads compute scaled dot-product attention across the sequence. This mechanism enables the network to assign higher weights to physiologically related PCG and SCG events, even when they occur at different time steps. Residual connections and layer normalization wrap this block to stabilize training and improve gradient flow.
- Position-wise feed-forward network: Linear transformations with an intervening rectified linear unit (ReLU) activation project each time-step embedding into a higher-dimensional space before returning it to the model dimension. Dropout (rate = 0.1) follows both the attention and feed-forward sublayers for regularization. Residual connections and layer normalization are also applied to preserve information and support deep optimization. These design choices facilitate gradient flow during training and stabilize deep-stack optimization, as established in the original transformer literature [29].

#### 3.3.2. ECG Reconstruction Decoder

The contextually refined sequence from the final transformer layer is passed through a time-distributed multi-layer perceptron (MLP) decoder. The MLP comprises two fully connected layers: a hidden layer with 128 units and ReLU activation, followed by an output layer that predicts a single value representing the ECG amplitude at each time step. This design enables the model to reconstruct the entire ECG waveform with the same temporal resolution as the input PCG–SCG segment.

The choice of an MLP for the decoding stage is guided by the characteristics of ECG signals. ECG waveforms exhibit both sharp, localized transitions, such as the QRS complex, and broader, smooth segments, such as the P and T waves. The MLP acts as a flexible regressor capable of mapping the temporally enriched features—learned through LSTM and transformer encoders—into precise amplitude predictions. Its simplicity allows it to avoid excessive temporal smoothing or phase distortion, which could obscure diagnostic features.

Moreover, the time-distributed structure of the MLP ensures that each time step’s output is generated based solely on its contextual embedding, preserving the modularity and interpretability of the output. This approach enhances adaptability to varying patient morphologies and supports clinically relevant waveform reconstruction for downstream analysis.

## 4. Experiments and Results

In this section, we describe the datasets, experimental procedures, evaluation metrics, and results for assessing the proposed hybrid LSTM-transformer model for electrode-free ECG reconstruction. We detail the public and custom datasets used for model training, the training protocols and data augmentation, the quantitative metrics for waveform similarity and peak alignment, and a comparative analysis of generated versus ground-truth ECG signals. We then present representative waveform results under different physiological conditions, highlight clinical case studies (e.g., arrhythmia episodes), and report ablation studies examining the contributions of each model component and the impact of transfer learning. Finally, we summarize the findings, discuss limitations such as inter-subject variability and signal bandwidth, and consider implications for practical deployment.

### 4.1. Datasets

(1) Public PCG–ECG datasets: To train the model to map mechanical and acoustic heart signals to electrical ECGs, we first trained on a public dataset containing synchronized PCG, SCG, and ECG recordings. The PhysioNet/CinC 2016 Challenge heart sound database was the second pre-training source. This corpus comprises 3126 PCG recordings from a variety of clinical and non-clinical environments [30]. Recordings range from a few seconds up to ~120 s, and each contains one PCG channel. Many of these recordings were acquired with a simultaneous single-lead ECG, although the official database files primarily emphasize heart sounds. We used the subsets that included aligned ECG, PCG, and SCG waveforms for training. To ensure consistent input dimensions, all signals from PhysioNet were resampled to 250 Hz before training. Preprocessing steps included notch filtering to remove powerline interference, baseline wander removal on ECGs, and normalization of waveform amplitudes. The datasets provided over 100 h of paired PCG, SCG, and ECG data for pre-training, covering a range of heart rates and acoustic conditions.

(2) Custom IoT sensor dataset: To adapt the model to mechano-acoustic-based vibration sensing, we collected a new dataset using a wireless IoT platform. Each subject wore a chest strap containing a three-axis MEMS accelerometer, a MEMS microscope sensor, and a reference single-lead ECG recorder. The accelerometer captured chest wall vibrations corresponding to cardiac mechanical activity, at a sampling rate of 250 Hz, with the ECG recorded synchronously at the same rate. This is analogous to prior work that recorded the chest accelerometer (SCG), heart sound (PCG), and ECG from multiple subjects. We enrolled 10 healthy volunteers (ages 15–50) to acquire records and recorded each under rest conditions—sitting quietly for 5 min. Accelerometer data were high-pass-filtered above 0.5 Hz to remove motion drift and bandpass-filtered in the 2–50 Hz range to isolate cardiac vibrations. A digital stethoscope recorded audio data in waveform audio file format (WAV), sampled at 48 kHz with 32 bits per sample. The ECG was bandpass-filtered (0.5–40 Hz), and all signals were normalized. The SCG, PCG, and ECG streams were time-aligned via simultaneous timestamps. This custom dataset, therefore, provides paired SCG, PCG, and ECG signals in realistic ambulatory scenarios. The dataset is representative of scenarios in which electrode-free monitoring could operate.

### 4.2. Experimental Procedure

#### 4.2.1. Signal Preprocessing and Network Implementation

**Figure 4:**
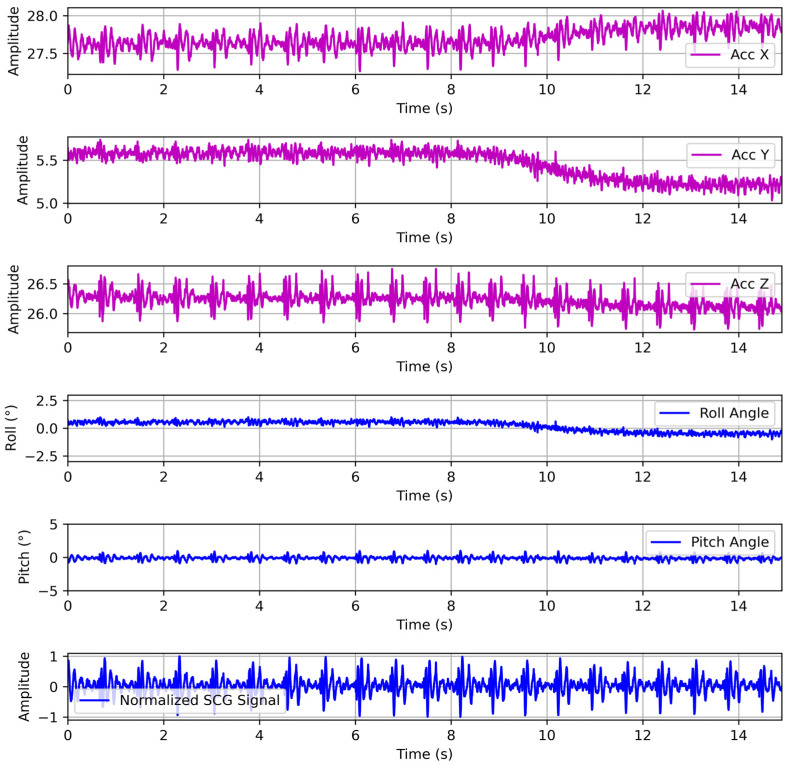
The raw three-axis accelerometer measurements, roll and pitch angles, and normalized SCG signal.

In our experimental procedure, the three-axis accelerometer measurements undergo a rigorous preprocessing pipeline to yield a normalized SCG signal that is robust to sensor orientation and gravitational artifacts. Each tri-axial sample is transformed into the world frame via the inverse rotation matrix. We used the full 3D orientation matrix to align the accelerometer frame with gravity. This geometric compensation removes the static gravitational component regardless of chest tilt and preserves only the true dynamic accelerations from cardiac and respiratory motion. Therefore, the gravity-compensated acceleration is bandpass-filtered between 2 and 50 Hz using a zero-phase finite impulse response filter to isolate SCG frequencies while suppressing low-frequency drift and high-frequency noise. After gravity subtraction, the dynamic accelerations with low-frequency drifts below ~0.3 Hz, which are caused by respiration, posture drift, or slow motion, remain. Therefore, we apply a high-pass filter with a 0.5 Hz cutoff to remove this residual baseline wander and ensure that the cardiac vibration signal returns to zero between beats. Filtered signals are then standardized by subtracting the segment mean and dividing by the segment standard deviation, ensuring amplitude normalization across subjects and recordings. To further enhance signal consistency, the normalized SCG is resampled to 250 Hz and aligned with the concurrently recorded ECG via timestamps. This preprocessing framework produces orientation-invariant, normalized SCG inputs that faithfully represent cardiac mechanical vibrations for subsequent deep learning–based ECG reconstruction. The raw three-axis accelerometer measurements, calculated roll and pitch angles without filtering, and normalized SCG signal are shown in Figure 4.

The proposed model uses a hybrid LSTM-transformer architecture to process SCG and PCG inputs. It begins with LSTM layers to encode temporal features from the time-series signals, including the peak and interval patterns of SCG and PCG. In practice, we used two-layer LSTMs with dropout applied after each recurrent layer to mitigate overfitting. The outputs of the SCG and PCG LSTM encoders are concatenated and fed into a shared transformer encoder. The transformer consists of multi-head self-attention layers with positional encodings to capture long-range dependencies and align features across modalities. Dropout is also applied between transformer layers. Finally, the fused feature is passed through a multi-layer perceptron decoder (with ReLU and a linear output) to predict the ECG waveform. Overall, this hybrid LSTM–transformer architecture combines LSTM’s strength in modeling sequential patterns with the transformer’s ability to capture a broader context. During training, we optimized this network with the Adam optimizer [31] (initial learning rate ~10^−3^, decayed over time) and minimized the mean squared error (MSE) between the predicted and true ECG signals. The MSE loss explicitly encourages waveform fidelity, as in prior ECG reconstruction work. We used 80% of the datasets for training, with a batch size of 64, and 20% for testing. In summary, the model has embedded LSTM layers for SCG and PCG, shared transformer layers for cross-modal fusion, dropout for regularization, and final dense layers to output ECGs.

**Figure 5:**
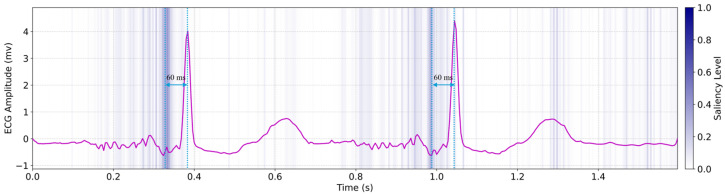
The saliency map of the hybrid LSTM–transformer model.

To interpret the underlying mechanisms of our deep learning model, we analyzed saliency maps generated by the hybrid LSTM–transformer architecture, revealing physiologically meaningful insights into the mechano–electrical coupling involved in PCG/SCG-to-ECG translation, as shown in Figure 5. Regions of high saliency (highlighted in dark blue) were consistently observed approximately 60 milliseconds prior to the QRS complexes in the predicted ECG. These high-saliency segments closely correspond to the timing of the PCG-derived first (S1) and second (S2) heart sounds, as well as SCG-detected aortic opening peaks [32]. The observed saliency shift reflects early mechanical cues, such as pre-stretch or atrial-induced ventricular filling, that preceded electrical activation. This pattern aligns with established electromechanical dynamics and suggests that the model captures subtle physiological features predictive of imminent cardiac electrical activity. Such temporally anticipatory saliency supports the model’s biological plausibility and highlights its reliance on interpretable mechanical markers for forecasting QRS onset. These findings are consistent with physiological studies of electromechanical timing in healthy cardiac function.

#### 4.2.2. Performance Analysis

We evaluated the model with simultaneously recorded ECG traces. This allows training on multimodal signals with PCG and SCG signals and the testing of ECG prediction. As shown in Table 1, we compared three modal settings for (1) an SCG-only baseline (identical architecture but trained using only the SCG input), (2) a PCG-only baseline (identical architecture but trained using only the PCG input), and (3) the proposed dual-modal hybrid LSTM–transformer model (trained on both SCG and PCG). All had the same architectures, except that absent inputs were set to zero. Performance was quantified by the RMSE of the reconstructed ECG waveform and the Pearson correlation coefficient between the predicted and true ECGs. These metrics are standard for assessing ECG reconstruction quality. The definition of the Pearson correlation coefficient (PCC) is as follows:

(5) $$PCC=\frac{\sum_{i=1}^{n}\left(x_{i}-\bar{x})(y_{i}-\bar{y}\right)}{\sqrt{{\sum_{i=1}^{n}\left(x_{i}-\bar{x}\right)}^{2}}\sqrt{{\sum_{i=1}^{n}\left(y_{i}-\bar{y}\right)}^{2}}}$$

where $x_{i}$ and $y_{i}$ are the two signals under comparison, and $\bar{x}$ and $\bar{y}$ are the two mean values of the signals. The root mean square error (RMSE) is defined in (6):

(6) $$RMSE=\sqrt{\sum_{i=1}^{n}\frac{{\left(x_{i}-{\tilde{x}}_{i}\right)}^{2}}{n}}$$

where $x_{i}$ is the ground truth value and ${\tilde{x}}_{i}$ is the predicted value.

Performance metrics are reported with 95% confidence intervals (CIs) calculated from t-distributions. The multimodal approach achieved a significantly lower RMSE (0.4868 ± 0.1631 mV, 95% CI) and higher correlation (0.9597 ± 0.0157, 95% CI) compared to single-modality baselines, as described in Table 1.

The SCG-only model displayed a higher RMSE and lower correlation than the PCG-only model, indicating poorer ECG reconstruction. This is because the PCG-only model benefits from high-contrast S1 and S2 sounds that align closely with QRS and T-wave events, resulting in a relatively low RMSE and high correlation. In contrast, SCG-only inputs are lower in amplitude and more prone to residual motion artifacts, making their vibrational peaks less distinct and leading to a substantially higher RMSE and lower correlation. In contrast, the dual-path model greatly reduced the RMSE and raised correlation, indicating much closer waveform matching. These trends are consistent with prior findings: single-modality mechanical-to-ECG models typically achieve moderate correlation, whereas including the acoustic PCG information improves alignment.

**Figure 6:**
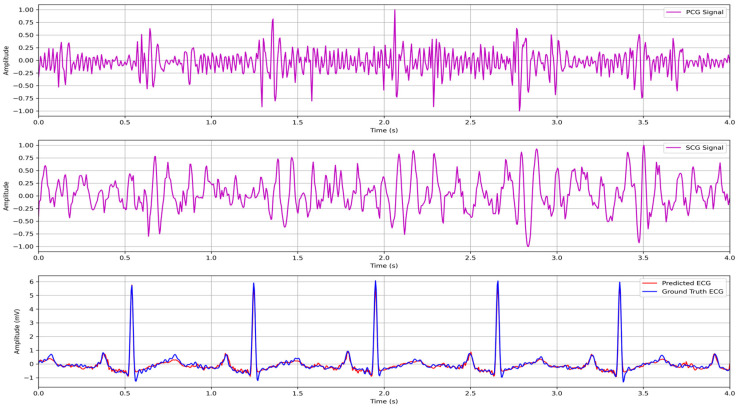
Reconstructed ECG wave with multimodal hybrid LSTM–transformer architecture.

Benefiting from multimodal training, our hybrid model achieved comparable or better correlations while using SCG or PCG only at inference. The reconstructed ECG with multimodal PCG and SCG is described in Figure 6.

**Figure 7:**
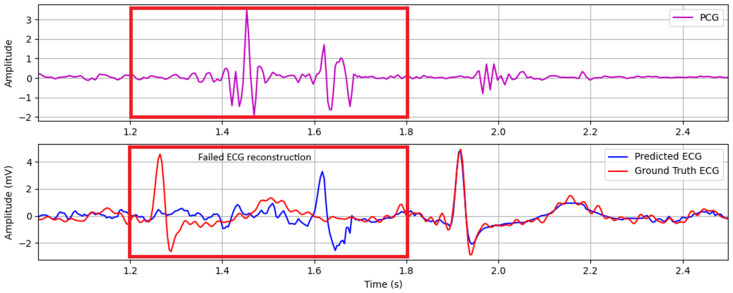
Failed ECG reconstruction caused by cough events. The red boxes marked the failed reconstruction period.

ECG reconstruction failures primarily stem from physiological interference sources, notably cough events and abdominal sounds originating from intestinal peristalsis. These low-frequency acoustic disturbances manifest as high-amplitude, gurgling waveforms that spectrally overlap with phonocardiogram components and corrupt mechano-acoustic feature extraction. This leads to erroneous ECG waveform generation. Figure 7 exemplifies this failure mechanism, demonstrating how cough-induced transient interference disrupts S1/S2 identification and consequently generates artifactual QRS complexes in the reconstructed ECG trace.

**Figure 8:**
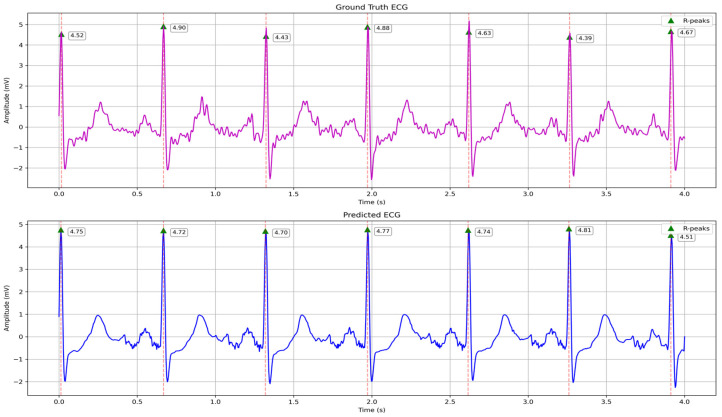
Comparison of R peaks between the ground truth and predicted ECG waves.

The accurate reproduction of R-peak amplitude is crucial for assessing ventricular depolarization strength in clinical monitoring. We detected R-peaks in both ground truth and predicted ECG traces by applying a standard amplitude threshold and local maximum search within each 1 s window. The ground truth and the model’s corresponding reconstructed R-peak amplitudes are shown in Figure 8. We computed the RMSE of these seven paired values as 0.2343 mV. This error corresponds to a mean amplitude bias of approximately 5.1% relative to the average ground truth R-peak (4.64 mV). The standard deviation of the amplitude differences is 0.219 mV. These results demonstrate that our hybrid LSTM–transformer model reliably restores the high-energy ventricular spikes essential for accurate heart rate and arrhythmia analysis.

This precision arises from the model’s multimodal design. A convolutional encoder extracts localized PCG and SCG features—capturing both valvular closures (S1/S2) and aortic valve opening/closure (AO/AC) vibrations—while residual LSTM blocks preserve temporal dynamics across cardiac cycles. Subsequent multi-head attention integrates long-range dependencies, aligning mechanical events with their electrical counterparts even when inter-beat intervals vary.

**Figure 9:**
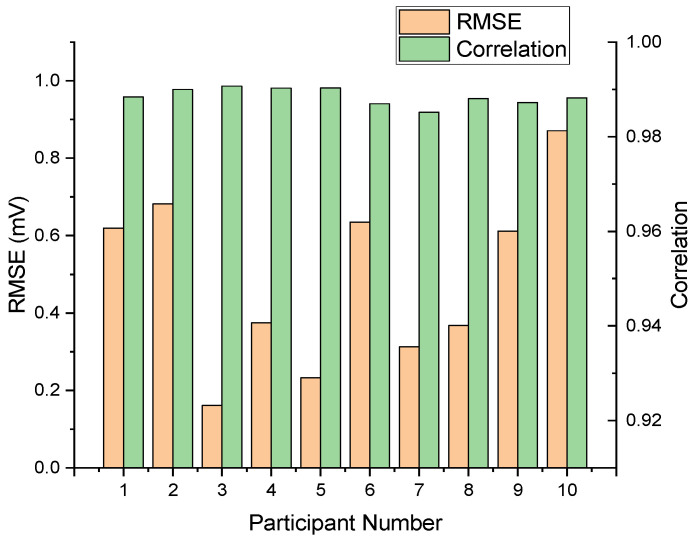
Performance analysis across 10 participants with RMSEs and Pearson correlation coefficients.

We assessed our multimodal LSTM–transformer model on data from ten participants, comparing reconstructed and reference ECG sequences. Figure 9 presents the per-subject root mean square error and Pearson correlation, yielding a mean RMSE of 0.4859 and a standard deviation of ±0.1961 mV. Pearson’s correlation coefficients ranged from 0.9186 to 0.9863, with a mean of 0.9653 and a standard deviation of 0.0218. These metrics demonstrate consistent waveform fidelity and temporal alignment across diverse users.

## 5. Discussion

Our results demonstrate that fusing PCG and SCG signals can effectively reconstruct the ECG waveform without the need for electrodes. The high correlation and low reconstruction error indicate the feasibility of this approach. In particular, the precise reproduction of the QRS complex suggests that heart rate and rhythm can be monitored reliably. Additionally, the system shows consistent performance across multiple healthy subjects, indicating robustness against inter-subject variability. These findings highlight the potential of mechano-acoustic fusion and deep learning to provide an accurate, electrode-free surrogate ECG based on the IoMT architecture.

However, several limitations must be addressed before broader clinical diagnoses. Firstly, current experiments were conducted at rest in healthy volunteers. We have not yet tested the system during physical activity or in patients with heart disease. Exercise and pathological conditions, such as valve narrowing or weakened heart muscle, can alter acoustic and vibrational patterns. Secondly, while our model accurately reproduces the ECG morphology (with a mean R-peak error of ~0.23 mV), clinical utility requires rigorous validation. Specifically, it is unclear how effectively the electrode-free ECG can detect arrhythmias or ischemic changes in comparison to conventional ECG systems.

To address these limitations, future work should focus on validating the system under diverse physiological and pathological conditions. This includes testing under different exercise conditions and across a spectrum of cardiac diseases. Moreover, rigorous clinical validation is essential to assess the diagnostic accuracy for detecting clinically relevant abnormalities. Prospective clinical trials will be necessary to establish the system’s performance and reliability in real-world healthcare settings.

## 6. Conclusions

We have presented an electrode-free approach to estimate the ECG waveform by fusing PCG and SCG signals with a hybrid LSTM–transformer network. Evaluated on both public and custom IoT datasets, the model achieved a mean Pearson correlation of 0.96 and an RMSE of 0.49 mV against clinical ECGs, significantly outperforming single-modality baselines. Our chest patch ESP32-based hardware with accelerometer and microphone sensors captures the mechano-acoustic signals and streams them for cloud processing. By eliminating the need for adhesive electrodes and conductive gels, this system reduces skin irritation and improves comfort for long-term cardiac monitoring.

Future investigations will focus on extensive clinical validation and system optimization. The method needs to be tested on cohorts with diverse cardiac pathologies and during dynamic activities. We will also optimize the model for efficient on-device inference, enabling fully wearable deployment. These advancements aim to strengthen diagnostic robustness and promote the widespread adoption of noninvasive cardiac monitoring solutions.

## Figures and Tables

**Table 1:** Quantitative comparison between multimodal and single-modal hybrid LSTM–transformer architectures.

Modal Mode	RMSE (mV) [95% CI]	Correlation [95% CI]
Multimodal: PCG-SCG	0.4868 ± 0.1631	0.9597 ± 0.0157
Single-modal: PCG	0.5909 ± 0.1952	0.9404 ± 0.0284
Single-modal: SCG	1.2716 ± 0.5090	0.7033 ± 0.1255

## Data Availability

Publicly available datasets were analyzed in this study. The PhysioNet/CinC 2016 Heart Sound Database is accessible at https://physionet.org/content/challenge-2016/1.0.0/ accessed on 11 June 2025.
